# Progress of Hydrogel Dressings with Wound Monitoring and Treatment Functions

**DOI:** 10.3390/gels9090694

**Published:** 2023-08-28

**Authors:** Shanshan Jin, Md All Amin Newton, Hongju Cheng, Qinchen Zhang, Weihong Gao, Yuansheng Zheng, Zan Lu, Zijian Dai, Jie Zhu

**Affiliations:** 1 School of Textiles and Fashion, Shanghai University of Engineering Science, Shanghai 201620, China; jinshanshan2021@126.com (S.J.); allaminnewton@sues.edu.cn (M.A.A.N.); 18051920601@163.com (H.C.); sxxxzhangqinchen@163.com (Q.Z.); gaoweihong@sues.edu.cn (W.G.); yuansheng@sues.edu.cn (Y.Z.); zanlu659@sues.edu.cn (Z.L.); 2 Innovation Center for Textile Science and Technology, Donghua University, Shanghai 200051, China

**Keywords:** wound monitoring, treatment, hydrogel, wound dressing

## Abstract

Hydrogels are widely used in wound dressings due to their moisturizing properties and biocompatibility. However, traditional hydrogel dressings cannot monitor wounds and provide accurate treatment. Recent advancements focus on hydrogel dressings with integrated monitoring and treatment functions, using sensors or intelligent materials to detect changes in the wound microenvironment. These dressings enable responsive treatment to promote wound healing. They can carry out responsive dynamic treatment in time to effectively promote wound healing. However, there is still a lack of comprehensive reviews of hydrogel wound dressings that incorporate both wound micro-environment monitoring and treatment functions. Therefore, this review categorizes hydrogel dressings according to wound types and examines their current status, progress, challenges, and future trends. It discusses various wound types, including infected wounds, burns, and diabetic and pressure ulcers, and explores the wound healing process. The review presents hydrogel dressings that monitor wound conditions and provide tailored treatment, such as pH-sensitive, temperature-sensitive, glucose-sensitive, pressure-sensitive, and nano-composite hydrogel dressings. Challenges include developing dressings that meet the standards of excellent biocompatibility, improving monitoring accuracy and sensitivity, and overcoming obstacles to production and commercialization. Furthermore, it provides the current status, progress, challenges, and future trends in this field, aiming to give a clear view of its past, present, and future.

## 1. Introduction

Skin is an organ covering the entire surface of the human body, one that is in direct contact with the external environment. It registers external stimulation, regulates body temperature, and protects the human body from external damage [1]. Being in direct contact with the external environment, the skin is a vulnerable tissue that is susceptible to trauma from injury or disease. Treating chronic wounds caused by bacterial infections poses a significant challenge in skin injury repair. The healing process of chronic wounds comprises four interrelated stages: hemostasis, inflammation, proliferation, and remodeling. Hemostasis involves the formation of clots to stop bleeding, followed by an inflammatory response that works to clear debris and combat pathogens. Proliferation entails the generation of new tissue, while remodeling involves the maturation and realignment of the healed wound. Understanding these sequential processes is crucial for developing targeted therapies to optimize wound repair and minimize complications [2]. However, the above methods generally cannot be carried out in a quick and orderly fashion, and various factors may lead to abnormal wound healing at any stage [3]. When problems with wound healing occur, infection can lead to delayed wound healing and even death [4]. Therefore, effective wound management is the key to promoting wound healing.

At present, researchers have successfully developed wound dressings with good self-healing, injectable, and antibacterial properties, such as sponges [5,6], freezing glue [7,8], membranes [9,10], aerogels [11,12], electro-spun scaffolds [13,14] and hydrogels [15,16]. Hydrogels are the preferred wound dressing material due to their excellent characteristics of hydropathy, biocompatibility, and resemblance to the extracellular matrix (ECM). Their hydrophilic nature allows the efficient absorption of wound exudate, while their three-dimensional pore structure supports cell migration, proliferation, and tissue regeneration. Hydrogels have, thus, become popular for wound management [17]. In recent years, hydrogel wound dressings fulfilling wound monitoring and treatment functions have become a research hotspot in the field of wound dressings because they can simultaneously assess the wound microenvironment and treat the wound on demand. Hydrogel wound dressings with built-in sensors or intelligent materials have been developed and offer a novel wound monitoring and treatment approach. These dressings utilize stimulus-responsive and self-healing materials to interact with the wound microenvironment, allowing the real-time sensing of wound conditions and any changes. By integrating sensors and intelligent materials, these hydrogel dressings enable the delivery of precise and targeted treatment interventions, based on the specific needs of the wound. This innovative technology holds promise for enhancing wound care by providing accurate monitoring and tailored therapeutic approaches for improved healing outcomes. Since the wound healing process is divided into different stages, and each phase has its unique microenvironment characteristics, the use of dressings to respond to changes in the wound microenvironment (such as temperature, pH, and blood sugar concentration) ensures that prompt and precise treatment can meet the treatment needs at each stage of wound healing. Thus, the goal of preventing infection, shortening treatment time, and reducing treatment costs can be achieved [18]. However, a comprehensive review of wound monitoring and therapeutic hydrogel wound dressings has still not been reported. This review provides an overview that first briefly introduces the preparation, properties, and applications of hydrogels, then discusses the types of skin wounds and the wound healing process, and subsequently delves into the realm of registered wound monitoring and therapeutic hydrogel dressings. These dressings’ current development status, progress, challenges, and prospects are thoroughly examined, emphasizing their role in wound monitoring and treatment. Furthermore, the review presents a forward-looking perspective by discussing the future development trends of hydrogel dressings that are equipped with wound monitoring and treatment functions. By encompassing these key aspects, this review aims to comprehensively understand the subject matter while highlighting the potential advancements and research opportunities of hydrogel wound dressings.

## 2. Preparation, Properties, and Applications of Hydrogels

Hydrogel is a highly hydrophilic gel with a three-dimensional network structure that can swell without dissolving in water [19,20,21]. Based on the hydrophilic chain segments (–OH, –COOH, and –NH_2_) in the polymer network, hydrogels have strong water absorption, swelling, and water retention abilities. Water molecules lose mobility; hence, hydrogels are also a kind of solid [22]. The preparation methods of hydrogels can be divided into chemical and physical cross-linking, according to their linkage mode [23]. Chemical cross-linking is achieved in the form of covalent bonds, while physical cross-linking is in the form of non-covalent bonds, such as ionic interactions, hydrogen bonds, or hydrophobic interactions. The polymers used to prepare hydrogels can be either natural, such as chitosan, carboxymethyl cellulose, sodium alginate, gelatin, etc., or synthetic, such as polyacrylic acid, polymethacrylic acid, N-isopropyl acrylamide, etc.

Wound dressings play a crucial role in safeguarding and fostering the healing process of injuries, encompassing trauma, burns, diabetic foot complications, and postoperative incisions. Hydrogels stand out among the various options as they fulfill most of the criteria for an ideal wound dressing. These criteria encompass absorbing surplus wound exudate, upholding a moist environment that encircles the wound, facilitating proper gas exchange, providing insulation, and delivering antibacterial properties. Additionally, they ensure safety, ease of removal from the wound’s surface, painless alteration during dressing changes, and simple application. These hydrogels also possess the necessary mechanical strength and viscoelastic attributes, including storage and loss modulus and suture retention strength, rendering them suitable for wound surface suturing or direct application to the wound area [24,25,26,27]. The hydrogel can form a semi-closed protective film at the injured area, providing a moist and breathable environment for healing and preventing the tissue from drying. At the same time, the hydrogel can absorb the wound exudate and promote the hydration of the wound, promote wound healing, and reduce the discomfort and possible infection of patients. Hydrogel dressings are often used in wounds such as pressure ulcers, local lesions of skin tissue, and tissue inactivation and are very effective for superficial wounds. Hydrogels can also be loaded with anti-inflammatory or bioactive substances that are slowly released in the vicinity of the patient’s wound, accelerating the healing process. The hydrogel has a particular mechanical strength, consistent with the biomechanical and viscoelastic properties (storage modulus and loss modulus, and the suture retention strength) of the wound surface or when applied to the wound [28,29,30,31,32]. In conclusion, a hydrogel dressing is an ideal wound dressing.

**Figure 1:**
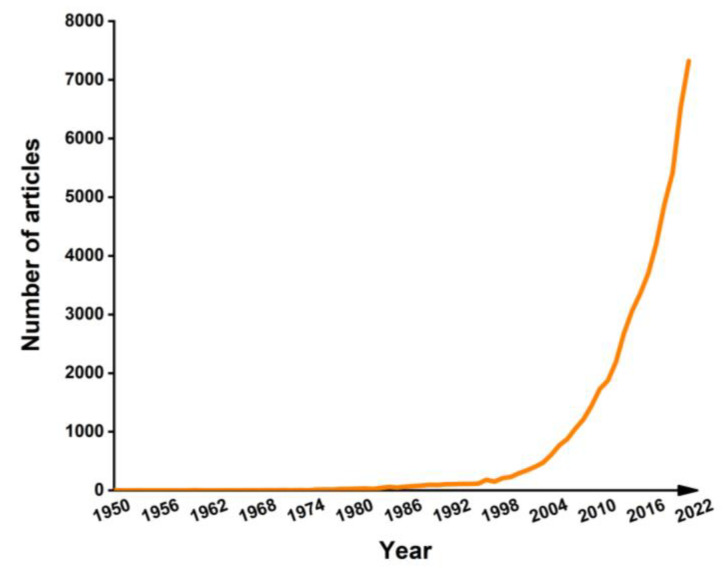
Temporal trend in published research articles on “hydrogels”, spanning the years from 1950 to 2022.

**Figure 2:**
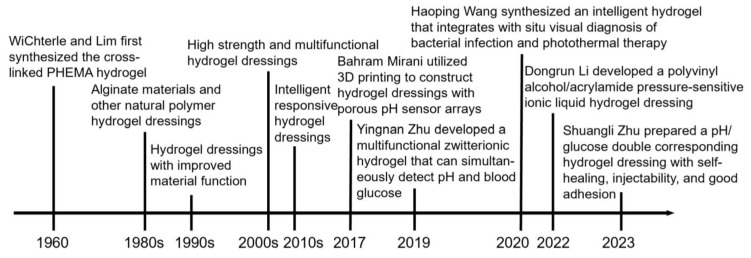
The evolutionary progression of hydrogels and dressings, along with contemporary hydrogel innovations for wound monitoring and treatment.

A search of the PubMed database for “hydrogel” revealed an increasing trend in the published articles [33]. Especially noteworthy is the literature’s substantial growth post-2004, as depicted in Figure 1. A comprehensive examination encompassing the creation, categorization, and utilization of all hydrogels would necessitate a more in-depth and specialized investigation exceeding the confines of this present review. The primary emphasis of these endeavors revolves around a thorough evaluation of the diverse facets associated with hydrogel dressings, featuring wound monitoring and treatment functionalities. Depicted in Figure 2 is a chronological progression of hydrogels, spotlighting the most recent advancements in hydrogel dressings with wound monitoring and treatment capabilities, as disclosed in this review article, spanning the period from 1950 to 2023 [34,35,36,37,38,39,40].

## 3. Types of Skin Wounds and Wound Healing

### 3.1. Overview of Skin Trauma

The skin is the primary physical barrier between the human body and the environment into which it comes in contact. It is crucial for thermoregulation and defense against foreign pathogens [41]. In general, the physiological structure of normal skin is divided into the epidermis and dermis. The epidermis layer, which is directly related to the outside world, mainly includes the keratinocyte layer and the germinal layer and has functions such as preventing tissue fluid outflow, anti-friction, and anti-infection. The dermis comprises dense connective tissue with papillary and reticular layers, from superficial to deep. Among them, the papillary layer of the skin is rich in capillaries, lymphatic vessels, nerve endings, tactile bodies, and other receptors, contributing to sensory perception and vascular supply. In contrast, the reticular layer predominantly comprises collagen, elastic, and reticular fibers, providing mechanical strength to the skin [42]. A wound is a defect or damage of the skin caused by external injury-causing factors (such as external force, surgery, thermal injury, chemical substances, etc.) or internal factors of the human body (such as physiological lesions of the body itself), which is usually accompanied by the breakdown of the structural integrity of the skin and the impairment of its functionality.

### 3.2. Types of Wound

There are numerous types of skin trauma, and wounds can be classified according to various methods [43]. Wounds can be classified into open wounds (such as abrasions, punctures, and lacerations) and closed wounds (such as crush injuries, contusions, blast injuries, and hematomas), depending on their location and exposure [44]. The wound’s depth can be divided into superficial wounds, partial cortical injury wounds, and entire cortical injury wounds. External wounds involving only the skin’s epidermis entirely heal within ten days. For some wounds that include cortical injury, the healing process is accompanied by scar formation and re-epithelialization, which basically takes 10 to 21 days. However, entire cortical injury wounds exhibiting damage to the dermis and subcutaneous tissue sites take longer to heal. According to the contamination status, wounds can be divided into clean, contaminated, and infected. The classification “clean wound” generally refers to a sterile surgical incision without contamination, such as the incisions made for liver and kidney surgery and thyroid surgery, or a blister that has not yet been contaminated, such as a wound formed by the removal of blister skin via a sterile operation in the complete blister of a second-degree scald. Contaminated wounds refer to injuries contaminated with bacteria that have not yet become infected, involving wounds of the digestive, respiratory, or reproductive systems, including acute trauma wounds. An infected wound is when the surrounding bacteria or pathogenic bacteria in the environment enter the body after the skin is damaged, causing infection. There is then inflammatory secretion at the wound, accompanied by local symptoms of swelling, heat, or pain [45].

Wound healing can be categorized into the healing of acute and chronic wounds, based on their healing time. Acute wounds are caused by sudden trauma and typically heal within a relatively short period. Conversely, chronic wounds are characterized by a delayed healing process that does not follow the standard and orderly repair sequence, resulting in the incomplete restoration of normal tissue. Various factors contribute to the development of chronic wounds, including conditions such as diabetic ulcers, venous ulcers, arterial ulcers, traumatic ulcers, and pressure ulcers. These wounds pose significant challenges due to their impaired healing potential and require specialized management strategies to promote successful wound closure [46]; chronic wounds significantly burden patients and the healthcare system [47]. Chronic wounds can be divided into infection wounds, burn injuries, diabetic ulcers, and pressure ulcers. They share standard features such as the upregulation of protease levels, elevated proinflammatory cytokines, excessive reactive oxygen species (ROS) levels, the persistence of senescent fibroblasts, prolonged infection, and stem cell dysfunction or insufficiency [48]. However, the characteristics of different types of chronic wounds and the underlying pathological mechanisms are diverse. This section will describe the features of several common types of chronic wounds.

#### 3.2.1. Infected Wounds

In chronic wound repair, a bacterial infection often occurs at the wound site. Once the wound is infected, bacteria will trigger persistent inflammation in the infected area, affecting the healing process or even causing the injury to fail to heal [49]. The clinical manifestations of infected wounds are erythema, edema, warmth, and the aggravation of pain. There is increased exudation or drainage from the wound and a growing stench. If the patient develops systemic symptoms, such as fever, chills, and leukocytosis, the infection will progress to bacteremia or sepsis [50]. Therefore, controlling wound infection is considered one of the most crucial challenges in bioengineering applications.

#### 3.2.2. Burn Wounds

Burns are one of the most common and devastating forms of wounds, and the evaluation of burn patients involves two key parameters: wound depth and total burn area. First-degree burns affect the superficial layer of the epidermis, while superficial second-degree burns involve the epidermis and dermis. Deep second-degree burns extend through the entire epidermis and dermis. Third-degree burns are the most severe, affecting the epidermis, dermis, and subcutaneous tissue. The classification of burns into these degrees aids in assessing the severity of the injury and guiding appropriate treatment strategies for optimal healing and recovery [51]. Second-degree and third-degree burns impair many vital functions of the epidermis and dermis. Severe burns, characterized by extensive tissue damage, can be life-threatening due to factors such as severe infection, hyperinflammation, reduced angiogenesis, insufficient production of the extracellular matrix, and inadequate stimulation of vascular growth factors (GFs). When the skin is affected by heat, rapid and dangerous fluid loss occurs in the body, along with condensation and the loss of proteins, including immunoglobulins, potentially leading to irreversible tissue damage and raising susceptibility to infection. In addition, cell membrane dysfunction can cause severe changes in the distribution of water and sodium in the body, while the loss of extracellular fluid and sodium consumption can further reduce blood volume and alter the electrolyte balance, leading to the death of burn patients [52,53,54].

#### 3.2.3. Diabetic Wounds

Diabetes mellitus, a prevalent metabolic disorder, has become a significant global health concern. Diabetic wounds exhibit distinct characteristics that are caused by hyperglycemia, chronic inflammation, hypoxia, inadequate vascularization, cellular infiltration, and fragile granulation tissue. These factors collectively impair the normal skin regeneration process, resulting in challenges for physiological wound healing. Consequently, treating diabetic wounds poses considerable difficulties in achieving successful closure and restoration. The intricate interplay of these pathophysiological factors underscores the need for specialized approaches to address the unique healing complexities associated with diabetic wounds. As a result, diabetic wounds take longer to heal than ordinary chronic wounds, and severe diabetic wounds, such as diabetic foot ulcers, can require amputation. Moreover, diabetic wounds often exhibit prolonged healing periods, with some cases persisting for an average duration of 12 to 13 months. Additionally, there is a general trend of diabetic wounds having a recurrence rate of 60 to 70%. However, it is essential to note that individual cases may vary regarding healing time and recurrence risk [55]. Treating diabetic wounds constitutes at least 12–15% of the total expenditure for diabetes treatment, contributing to 40% of the national healthcare costs. The complex pathogenesis, pathogen invasion, and high incidence increase the cost and difficulty of treatment and seriously affect the comfort and health of patients [52,56,57,58]. Treating diabetic wounds is particularly difficult since the wound requires the highly ordered and continuous residency and recruitment of cells, GFs, and cytokines to facilitate healing [59].

#### 3.2.4. Pressure Ulcers

Pressure ulcers, also known as pressure sores, usually occur in areas where the bones protrude, such as the sacrum (the base of the spine), buttocks, and heels [60]. Pressure ulcers are caused by prolonged pressure, friction, or shear forces that impair the blood supply to the affected area and cause tissue malnutrition. This type of pressure ulcer is common in patients with reduced mobility, paralysis, coma, or the long-term bedridden. In such patients, it is not possible to release pressure areas adequately. The prolonged exposure of an area of the body to pressure interrupts local blood circulation and triggers a series of biochemical changes that may lead to tissue damage and ulceration [50]. According to the severity of the wound, pressure ulcers can be divided into four stages. The first stage is congestion and redness, mainly in the form of local skin swellings, pain, and numbness. The second stage is the inflammatory infiltration stage; the skin will turn purple, and the affected area has induration and is accompanied by pain. Stage three is the superficial ulcer stage, where the affected area will be ulcerated, and the subcutaneous tissue will be exposed. Stage 4 is the necrotic ulcer stage; patients will develop symptoms of necrosis in the affected area, and this may even cause sepsis. Pressure ulcers that cannot be staged in this way are typically characterized by a full-thickness loss of tissue, a covering of the decaying flesh at the base of the ulcer (yellow, tan, gray, green, or brown), or an eschar attachment to the wound bed (carbon, brown, or black) [61]. The patients themselves experience malnutrition, long-term bed rest, and even paraplegia, along with some nerve loss, leading to difficult wound healing. Some patients are also in difficult economic situations; therefore, it is difficult to heal the wounds of such patients with pressure ulcers. In the early stages, systematic and standardized treatment plans should be formulated, including strengthening the nutrition of patients, with sequential treatment and the treatment of wounds.

### 3.3. The Process of Wound Healing

**Figure 3:**
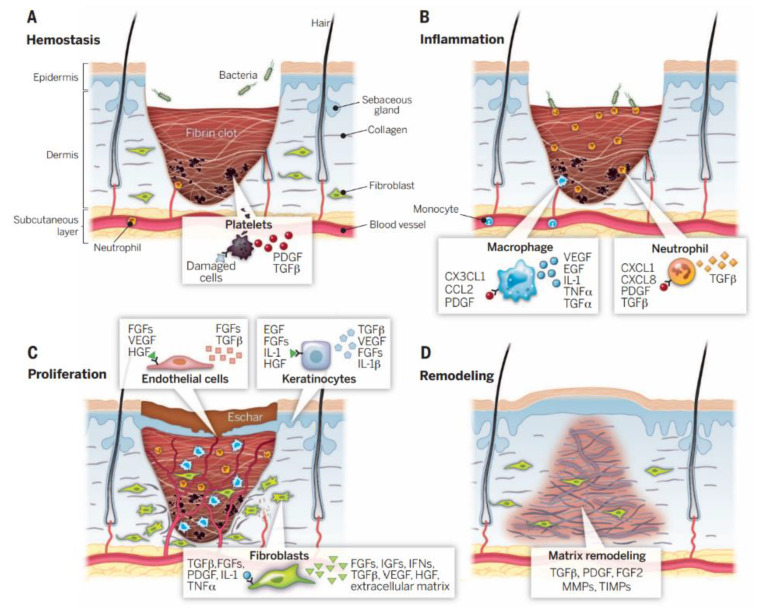
Wound healing is a complex process that can be broadly divided into four sequential stages: (**A**) hemostatic stage, (**B**) inflammatory stage, (**C**) proliferative stage, and (**D**) remodeling stage [64].

When skin tissue is injured, the body’s immune system immediately initiates a cascade of chemical signals between the different tissue cells, including immune function cells. At the same time, human biological signal-triggering molecules will immediately start the subsequent wound repair process [62]. As shown in Figure 3, the healing process of chronic wounds is mainly divided into four stages, namely, the hemostasis stage, the inflammation stage, the tissue proliferation stage, and the tissue remodeling stage [63].

During the hemostasis phase, tissue coagulation is triggered immediately after skin trauma. Platelets in the blood components come into contact with exposed collagens and other elements of the body’s natural extracellular matrix. This contact quickly triggers the release of platelet coagulation factors, after which many endothelial cells gather. The dynamic balance between platelet-induced coagulation and fibrinolysis collaborates to regulate the hemostatic responses, vasoconstriction, and the exudation of blood and tissue fluid [65,66]. During the inflammatory phase, white blood cells will enter the wound area from the capillaries around the wound tissue and absorb a large amount of tissue-inflammatory substances. The inflammatory cells will then release a large amount of growth factors. The signal factors will immediately stimulate macrophages and other immune cells and will continue to migrate to the wound to phagocytose cell debris. The injury will appear red, swollen, hot, and painful, in a pathological phenomenon [43]. In the stage of tissue proliferation, along with the migration of fibroblasts, new ECM tissue structures are continuously synthesized at the wound site, and a large amount of ECM accumulation will further promote cell migration [67]. Finally, during the stage of tissue remodeling, the newly generated collagen matrix becomes more directional, and new epithelial tissue and scar tissue gradually form [68].

## 4. Hydrogel Dressings with Wound Microenvironment Monitoring and Treatment Functions

Wound healing is a complex process that is influenced by changes in the wound microenvironment, including acid and alkaline levels, temperature, blood glucose, and pressure. Developing a hydrogel dressing system with intelligent monitoring and dynamic treatment functions is significant. This system would enable the collection of wound parameters and provide targeted interventions during healing. Integrating monitoring and treatment capabilities in a hydrogel dressing can enhance wound healing outcomes by offering personalized and precise interventions that are based on specific wound needs. This integration represents an important advancement in the field, allowing for real-time parameter collection and targeted treatment within a hydrogel dressing system [69]. This part of the paper will review pH-sensitive, temperature-sensitive, glucose-sensitive, and pressure-sensitive hydrogel dressings, and their research progress will be introduced. 

### 4.1. pH-Sensitive Hydrogel Dressing

**Figure 4:**
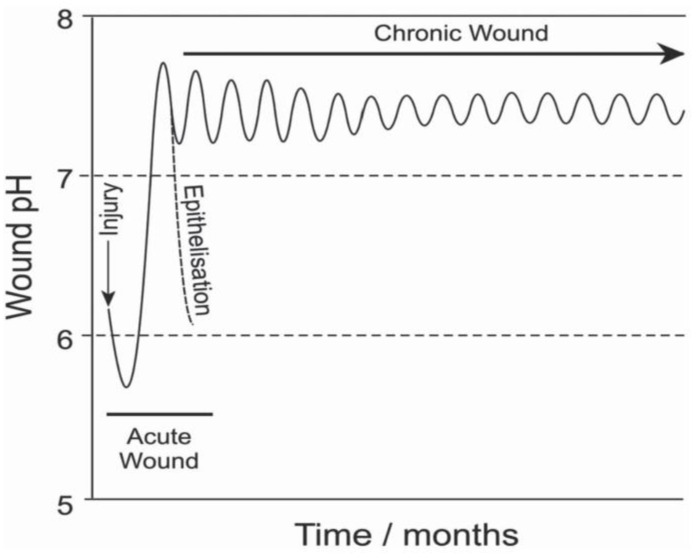
The pH value in the wound microenvironment changes with time [72].

The pH of healthy skin is slightly acidic, with a range of 4.8 to 5.7, showing a weakly acidic appearance [70]. When skin is damaged, exudate will appear at the wound site, and the pH value of the exudate will change along with a series of pathological changes, such as inflammation, collagen formation, and angiogenesis. In the inflammatory stage, the contraction of blood vessels in the local tissue of the wound will cause insufficient blood supply, resulting in a lack of nutrition and oxygen. At the same time, glycolysis leads to increased lactic acid and CO_2_, eventually leading to a decreased pH. If infection is present in the wound, the pathogen breaks the extracellular matrix and produces ammonia, which makes the wound alkaline. Without the corresponding intervention measures, neutrophils will rapidly aggregate and release excessive elastase, which will interact with necrotic and inactivated tissues in the wound, leading to increased metabolic load at the injury and prolonged wound healing. An acidic environment can increase the oxygen supply, induce fibroblast proliferation, and facilitate wound healing. Clinical studies have shown that the pH value of chronic wounds can range from 7.15 to 8.90, providing an alkaline environment that can support the growth and proliferation of certain bacteria. For example, the pH value for the survival of *Staphylococcus aureus* is 7.0–7.5 [71]. Therefore, when a wound is infected, one of the essential indicators of the wound microenvironment is an increase in pH (as shown in Figure 4) [72].

**Figure 5:**
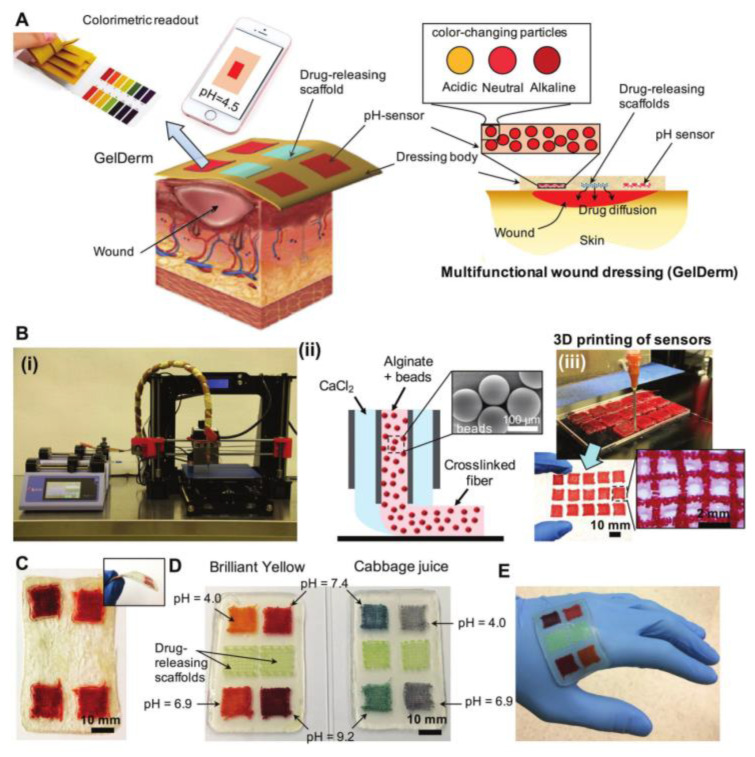
An advanced multi-purpose dressing for wound monitoring and management. (**A**) Schematic representation of dressing treatment of epidermal wounds, with pH-sensitive and drug-eluting components. (**B**-**i**) Porous sensors were fabricated using a 3D bioprinter equipped with a co-axial flow microfluidic nozzle. (**B**-**ii**) Schematic of fiber deposition using the co-axial flow system. (**B**-**iii**) 3D printer can be programmed to produce arrays of porous sensors for fabrication of large-scale dressings. (**C**) Dressings can be lyophilized and sterilized for storage and transportation. (**D**) Synthetic Brilliant Yellow and naturally derived cabbage juice were used as model pH indicators for the fabrication of the sensors. Sensor arrays enable detecting spatial variations of pH on the wound site. Drug-eluting scaffolds release high doses of antibiotics at the wound site to eradicate the bacteria that may remain on the wound site each time the dressing is replaced. (**E**) The multi-purpose dressing can maintain a conformal contact with irregular surfaces [36].

Researchers mainly use the colorimetric response method to prepare pH-sensitive hydrogel dressings. The specific process used is to sense the acid-base microenvironment of the wound by adding pH discoloration materials to the dressing, producing a color change. Bahram Mirani et al. [36] incorporated pH-responsive color-changing mesoporous resin beads into alginate fibers. They utilized 3D printing to construct hydrogel dressings with porous pH sensor arrays, as illustrated in Figure 5. This innovative approach enables real-time pH monitoring within the hydrogel dressings, facilitating precise interventions for optimal wound healing. This developed hydrogel dressing provided real-time wound data, such as the degree of bacterial infection and antibiotic release, through color changes. The dressing enabled digital remote diagnosis and treatment when it was connected to an image acquisition device. This technology enhances wound care by offering comprehensive monitoring and interventions based on accurate and timely wound information. Furthermore, this dressing demonstrated non-toxicity upon contact with human primary keratinocytes and fibroblasts. This attribute positions it as a viable option for addressing dermal injuries effectively and safely. However, considering that there may be too little or too much wound exudate during clinical use, or the exudate itself may be discolored (in bloody, suppurative wounds, etc.), the clinical promotion of this dressing is still being promoted.

**Figure 6:**
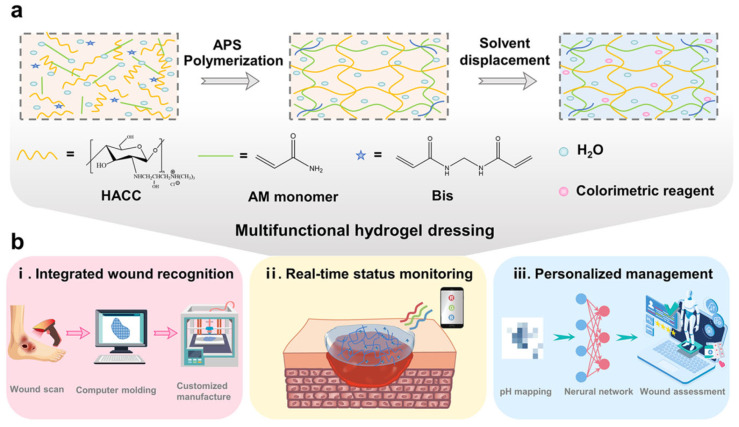
Schematic representation of multifunctional hydrogels for wound management. (**a**) Illustration demonstrating the preparatory steps involved in the synthesis of multifunctional hydrogels. (**b**) Schematic diagram showcasing multifunctional hydrogels’ intelligent wound monitoring capabilities when employed as wound dressings. This includes the processes of wound recognition, real-time condition monitoring, and personalized wound management [73].

Lirong Wang et al. [73] developed a multifunctional hydrogel wound dressing by incorporating colorimetric reagent litmus into a hydrogel that is composed of polyacrylamide and the quaternary ammonium salt of chitosan (HACC-PAM). The preparation process and working principle of this multifunctional hydrogel are illustrated in Figure 6. By utilizing a smartphone, the chromaticity signal is converted into a pH-sensing image. The color variation between the wound edge and normal skin is then used to generate pixel cloud data for wound image generation. A convolutional neural network machine-learning algorithm is employed to provide personalized wound management feedback, based on a wound management model. The pH distribution of the wound is analyzed using the colorimetric signal of the hydrogel, enabling the evaluation and prediction of wound healing and infection status. The intelligent wound monitoring process comprises wound recognition, real-time status monitoring, and personalized wound management. Online wound scanning and offline intelligent printing enable the accurate fitting of irregularly shaped wounds with the prepared wound dressing. This hydrogel dressing has the potential to help prevent or reduce the risk of bacterial infection due to its properties and design. The Cell Counting Kit-8 (CCK-8) assay and the live/dead staining method were used to investigate the viability of fibroblast cells (NHDF) that were exposed to the multifunctional hydrogel. The living cells co-cultured with the hydrogel were indistinguishable from the control group, which were spindle-shaped, and the cell density increased considerably with the duration of culture time. These results confirmed the excellent biocompatibility of these multifunctional hydrogels for wound dressings. Moreover, resource waste is caused by wound exposure or excessive coverage, whereas this procedure successfully realized the integration of wound identification and the precise matching of treatment by accurately locating the active ingredients in the wound. This hydrogel dressing could monitor the state and progression of wound infection, providing valuable information about the presence and severity of the infection, but the solvent replacement method used in the preparation of hydrogels is time-consuming, which is not conducive to their clinical promotion and application.

**Figure 7:**
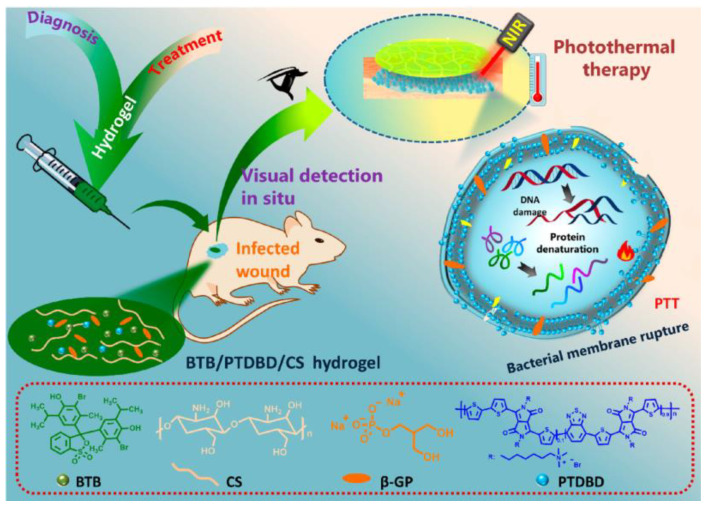
Diagram depicting the hydrogel microenvironment: BTB/PTDBD/CS—Enabling responsive naked-eye diagnosis and photothermal treatment for wound infection [38].

Haoping Wang et al. [38] synthesized an intelligent hydrogel that was integrated with the in situ visual diagnosis of bacterial infection and photothermal therapy, as shown in Figure 7. The synthesized BTB/PTDBD/CS hydrogel, achieved by incorporating pH-sensitive bromothymol blue (BTB) and near-infrared absorption conjugated polymer (PTDBD) into a heat-sensitive chitosan (CS) hydrogel, demonstrated the potential for diagnosing the acidic microenvironment of *Staphylococcus aureus* biofilms and infected wounds by visualizing color changes. This hydrogel enabled rapid diagnosis, allowing immediate local hyperthermia of the infected site when subjected to near-infrared laser (808 nm) irradiation. Moreover, it offers the potential for treating refractory biofilms that are challenging to eradicate. The unique combination of BTB, PTDBD, and CS within the hydrogel provides a promising approach for diagnosing and treating infected wounds, facilitating efficient and targeted therapy. This hydrogel will guide the development of intelligent and convenient platforms for diagnosing and treating bacterial infections, but there are still problems, such as imprecise monitoring ability and the easy leaching of dyes. Similarly, Asmaa Ahmed Arafa et al. [74] employed the pH-sensitive natural dye, Curcuma longa extract (CLE), as a cost-effective and straightforward pH-sensitive indicator, which was loaded into a hydrogel composed of hydroxyethyl cellulose grafted with itaconic acid. This preparation yielded a transparent, soft, and pH-sensitive wound dressing. Experimental findings indicated that the material exhibited a distinct color change when the hydrogel matrix was applied to cotton yarn and then exposed to various pH buffer solutions. These results highlight the potential of a pH-sensitive hydrogel dressing for the visual detection and monitoring of pH changes in wound environments. When the pH was ≤7, the dressing appeared yellow; it changed to dark red when the pH was >7. This wound dressing offers the therapeutic effect of CLE and can visually display the wound’s pH value. However, it also has some problems, such as inaccurate monitoring ability, the easy leaching of dye, and poor wound healing effects.

### 4.2. Thermo-Sensitive Hydrogel Dressing

**Figure 8:**
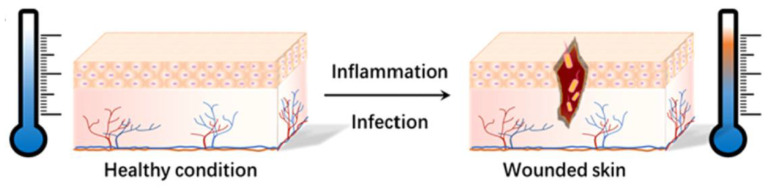
The body temperature rises when a wound becomes infected or inflamed [77].

For chronically infected wounds, chronic burn wounds, and pressure sores, the temperature of the injury will also differ from that of the intact tissue, despite changes in the pH of the injury. Body temperature is average (36 to 37.5 °C) when the skin is healthy but increases when infection or inflammation develops in the wound (Figure 8). Both internal physiological factors and external environmental conditions, including ambient temperature, surface moisture, and body position, influence skin temperature. In wound infection, elevated temperatures are observed due to the body’s immune response, comprising vasodilation, induced by inflammatory cytokines, and increased tissue metabolism. It is worth noting that near-normal temperatures within the wound environment promote cellular division and facilitate optimal wound-healing processes. The interplay between temperature regulation and wound healing underscores the importance of monitoring and maintaining the appropriate temperature conditions for effective wound management. An increase in temperature at any point after surgery can indicate wound infection. However, the timing of temperature changes can vary, depending on factors such as the type of surgery and individual patient characteristics [75]. In a clinical context, the skin temperature difference between a specific target site and a symmetrical contralateral reference point is usually used as a reference. The presence or absence of infection in the wound in one study was determined using validated assessment tools and clinical judgment [76]. To this end, researchers have developed temperature-sensitive and thermally responsive hydrogel dressings to monitor the state of the wound and deliver drugs and factors that promote wound healing [77].

**Figure 9:**
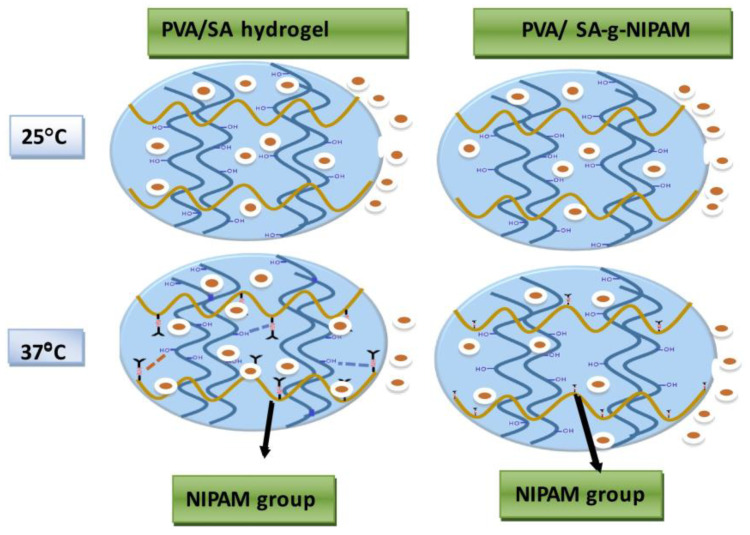
Drug release from PVA/SA-g-NIPAM hydrogel dressings at different temperatures [80].

For burn wounds, hydrogel dressings with temperature-responsive drug release systems have been developed. Min Hee Kim et al. [78] synthesized a thermo-sensitive methylcellulose hydrogel containing nano-silver oxide using a one-pot method, by taking advantage of the salting-out effect of silver acetate precursor (CH_3_COOAg) in methylcellulose solution. At the same time, silver nanoparticles have excellent antibacterial activity to promote the healing of burn wounds. However, the development of this hydrogel dressing was primarily focused on the treatment aspect of burn wounds, rather than on monitoring the wound microenvironment. In addition, the preparation of thermosensitive hydrogels using isopropyl acrylamide (NIPAM) monomers has been widely reported because the minimum critical solution temperature of NIPAM monomers is approximately 32 °C, which is close to the physiological temperature [79]. A.S. Montaser et al. [80] developed a hydrogel using alginate grafted with N-isopropyl acrylamide (NIPAM) and polyvinyl alcohol. The hydrogel was further encapsulated with the anti-inflammatory drug, diclofenac sodium (DS). Notably, the release of DS from the hydrogel occurred in distinct stages at different temperatures, as depicted in Figure 9. At approximately 25 °C, the drug exhibited continuous release from the hydrogel. However, when the temperature increased to 37 °C, a second phase of drug release was observed, which was attributed to the temperature-responsive behavior of NIPAM within the hydrogel; this temperature-dependent release mechanism demonstrates the potential of the developed hydrogel for controlled and targeted drug delivery, particularly in response to changes in temperature. This temperature-triggered stepwise drug release method shows the feasibility of preparing thermosensitive hydrogel dressings. However, thermo-sensitive wound dressings require proper contact with the wound site to sense temperature changes accurately, enabling them to exhibit their thermosensitive properties and provide reliable temperature data.

**Figure 10:**
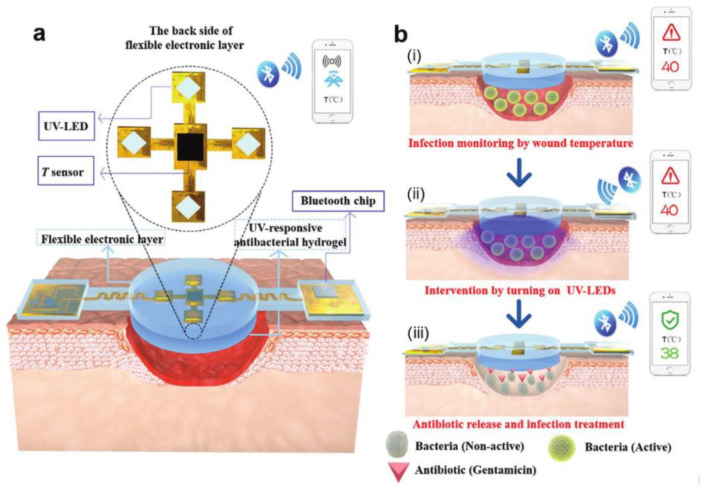
Schematic diagram of the structure and working principle of intelligent, flexible, electronic integrated wound dressing. (**a**) The integrated system consists of a polydimethylsiloxane-encapsulated flexible electronic layer and an UV-responsive antibacterial hydrogel. The flexible electronic device is integrated with a sensor for monitoring temperature and four UV-LEDs for emitting UV light (365 nm) to trigger the release of antibiotic from the UV-responsive antibacterial hydrogel when needed; a Bluetooth chip is also integrated for wireless data transmission in real time. (**b**) Conceptual view of the integrated system for infected-wound monitoring and on-demand treatment: (**i**) real-time monitoring of wound temperature and providing an alert of hyperthermia caused by infection; (**ii**) turning on UV-LEDs to trigger the release of antibiotics; (**iii**) infection inhibition by the released antibiotics, resulting in decreased wound temperature [81].

When thermo-sensitive hydrogel dressings are connected to image acquisition devices, the temperature sensitivity of wound dressings is improved, and digital remote diagnosis and treatment can be realized. Qian Pang et al. [81] designed an intelligent, flexible electronic integrated wound dressing composed of flexible electronic components, temperature sensors, UV light-emitting diodes in the upper layer, and UV-responsive antibacterial hydrogel in the lower layer. The developed dressing incorporated integrated sensors to enable the real-time monitoring of wound temperature, serving as an early predictor of pathological infection. Notably, the drug release from the heat-responsive vector within the dressing was triggered when a maximum temperature of 40 °C persisted for more than 6 h, which indicates the presence of infection. This integrated system demonstrates the efficacy of real-time monitoring, early diagnosis, and controlled drug delivery, as illustrated in Figure 10. The wound dressing in this study possessed numerous desirable characteristics, including flexibility, compatibility, high monitoring sensitivity, and durability, making it a promising solution for wound management. The assessment of hydrogel dressing cytotoxicity involved evaluating the viability of NIH 3T3 cells, cultured using the extraction medium. With increased culture time, the viabilities of the NIH 3T3 cells exhibited an upward trend across all groups. This trend suggests that the hydrogel dressing did not exert any adverse effects on the proliferation of NIH 3T3 cells. This way of combining hydrogels with temperature sensors to monitor the wound microenvironment and dynamically deliver drugs according to real-time needs represents the development direction of a new generation of intelligent sensors. However, due to the high cost and short service life of the response elements, most can only be used as disposable dressings.

**Figure 11:**
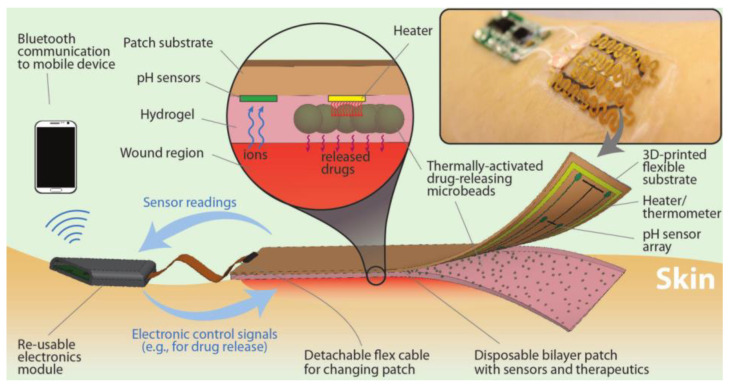
Schematic illustration of an automated smart bandage [82].

By integrating temperature and pH sensors into a flexible bandage, Pooria Mostafalu et al. [82] designed a flexible, intelligent, and automated wound dressing, as depicted in Figure 11, with the aim of real-time wound status monitoring. The dressing incorporated a stimulus-responsive system comprising a hydrogel loaded with a thermosensitive drug carrier and an electronically controlled flexible heater to facilitate on-demand drug delivery. The dressing also featured a microcontroller that processed sensor data and enabled personalized treatment by programming the drug release protocol. The flexible wound dressing was attached to the precisely shaped medical tape to ensure practicality and comfort, resulting in a wearable material of less than 3 mm thick. The design emphasizes the cost-effectiveness and disposability of the sensing module and the integrated heater, while the electronics are designed for reusability. This intelligent, flexible wound dressing offers promising personalized and efficient wound management capabilities.

### 4.3. Blood Glucose-Sensitive Hydrogel Dressing

For diabetic wounds, hyperglycemia can lead to changes in the cellular microenvironment around the wound, including a series of complex changes. These detrimental effects include a significant reduction in the macrophages, the accumulation of advanced glycation end products, and the production of reactive oxygen species (ROS), which lead to the weakening of wound resistance and proliferation ability. In addition, high blood glucose will also make the blood glucose concentration around the wound higher, causing bacteria to breed and making the wound more susceptible to infection after the injury [83].

**Figure 12:**
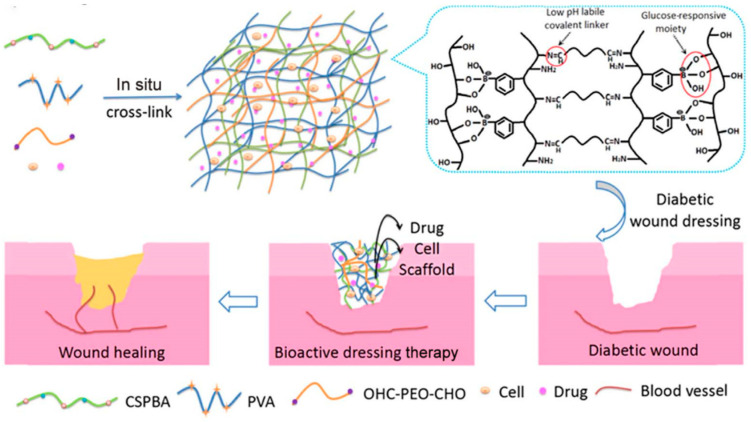
The hydrogel wound dressing is made with a Schiff base and phenyl borate ester bond [84].

To address the specific needs of diabetic wounds that are associated with high blood glucose levels, it is essential to develop hydrogel wound dressings that respond to changes in blood glucose concentration. Lingling Zhao et al. [84] successfully established a glucose-triggered drug release system using a Schiff base and the phenyl borate reaction, as depicted in Figure 12. The hydrogel developed in this study exhibited responsiveness to pH and glucose levels as a proof of concept; insulin and fibroblasts were employed as model drugs and cells, respectively. The insulin-fibroblast dual-loaded smart hydrogel was utilized as an active dressing for treating diabetic ulcer wounds. Since the Schiff base bonds are unstable in an acidic environment, they can easily be hydrolyzed, leading to the bonds breaking. The phenyl borate binds glucose more quickly than the hydroxyl group; therefore, lowering the pH in the wound microenvironment or increasing the glucose level will lead to the hydrolysis of the Schiff base bonds in the hydrogel matrix and accelerate the release of insulin. As high glucose levels are characteristic of chronic diabetic ulcer wounds, this hydrogel dressing was designed to respond to the specific needs of diabetic wounds associated with elevated glucose levels. Moreover, due to the sustained release of insulin in the hydrogel and the hypoglycemic function of chitosan, the blood glucose level at the wound site was controlled. The glucose-responsive wound dressing developed in their study offers benefits beyond wound healing by actively contributing to regulating blood glucose levels. This innovative approach presents a new perspective on wound healing and diabetes treatment. By responding to changes in glucose levels, the dressing not only aids in accelerating wound healing but also provides a means of managing blood glucose levels. Furthermore, the reported cell viability and proliferation experiment display the good viability of the cells cultivated in the hydrogel matrix. Shuangli Zhu et al. [40] prepared a composite hydrogel with self-healing capabilities, good injectability, and adhesive properties by the dynamic phenyl borate ester bonding of dopamine-modified gelatin and phenylboronic acid-modified hyaluronic acid, along with the incorporation of metformin and copper-loaded dopamine nanoparticles into ECM-simulated hydrogel. The release of metformin from the hydrogel dressing demonstrated suitable pH and glucose responsiveness, enabling the intelligent treatment of diabetic wounds. This responsive drug delivery mechanism allowed metformin’s targeted and controlled release in response to specific pH and glucose conditions within the wound environment. By leveraging this intelligent drug release system, hydrogel dressings offer a promising approach for the tailored and effective treatment of diabetic wounds.

**Figure 13:**
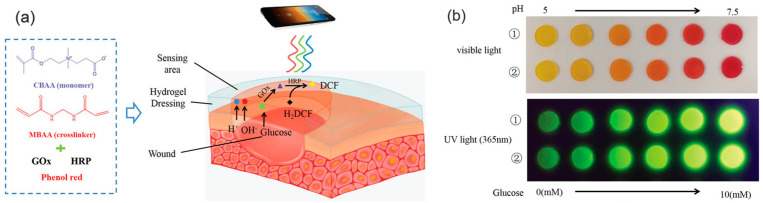
Visualization of a zwitterionic PCB hydrogel dressing, coated with a pH indicator (phenol red) and glucose-sensitive enzymes (GOX and HRP), for pH and glucose concentration detection in wound exudate. (**a**) Scheme of PCB hydrogel dressing for the detection of pH value and glucose concentration in wound exudate. (**b**) Functionalized wound dressing for simultaneous detection of pH values (under visible light) and glucose concentrations (under UV light) [37].

High blood glucose levels can impair the normal wound-healing process by reducing the oxygen and nutrient supply to cells, resulting in a significantly slower healing rate of diabetic wounds. Therefore, some researchers have loaded glucose oxidase (GOx) to lower blood glucose around the wound. Yingnan Zhu et al. [37] developed a multifunctional zwitterionic hydrogel to simultaneously detect two dynamic wound parameters, namely, pH and blood glucose levels, to monitor the status of diabetic wounds, as illustrated in Figure 13. This hydrogel system encapsulated a pH-indicating dye (phenol red), along with two glucose-sensitive enzymes, glucose oxidase (GOx) and horseradish peroxidase. These components were incorporated into a bio-repellent and biocompatible zinc–ionic polycarboxy betaine alkali hydrogel matrix. The wound parameters were quantified by analyzing an RGB signal that was converted from visual images. This multifunctional wound dressing could effectively monitor and detect pH changes within a specific range that is relevant to wound healing, such as a pH of 5 to 7 and blood glucose levels ranging from 0.10 to 10 × 10^−3^ mol/L. This type of multifunctional wound dressing is expected to open up the prospect of chronic wound management and guidance for the clinical use of diabetes. Based on zwitterionic carboxy betaine, Hongshuang Guo et al. [69] developed a multi-responsive zwitterionic skin hydrogel sensor system (SB-N-MB) by combining temperature-sensitive N-isopropyl acrylamide (NIPAM) with glucose-sensitive methyl acrylamide phenyl boric acid. The resulting sandwich structure enabled continuous real-time monitoring and the differentiation of temperature, glucose concentration, and wound strain. This innovative sensor system facilitated wound monitoring and showed the potential of promoting the healing of diabetic wounds. The zwitterionic skin-sensing system holds significance in wound management and opens new avenues for artificial intelligence by providing a platform for multiple signal discrimination.

### 4.4. Pressure-Sensitive Hydrogel Dressing

Pressure ulcers pose significant challenges in healthcare settings, leading to prolonged hospital stays, severe pain, and increased mortality rates. This places immense pressure on both patients and medical institutions. As a result, preventing and treating pressure ulcers in long-term bedridden patients have become urgent issues to address. Researchers have developed multifunctional wound dressings that integrate pressure sensing, real-time monitoring, and wound treatment capabilities to address secondary pressure injuries sustained during treatment.

**Figure 14:**
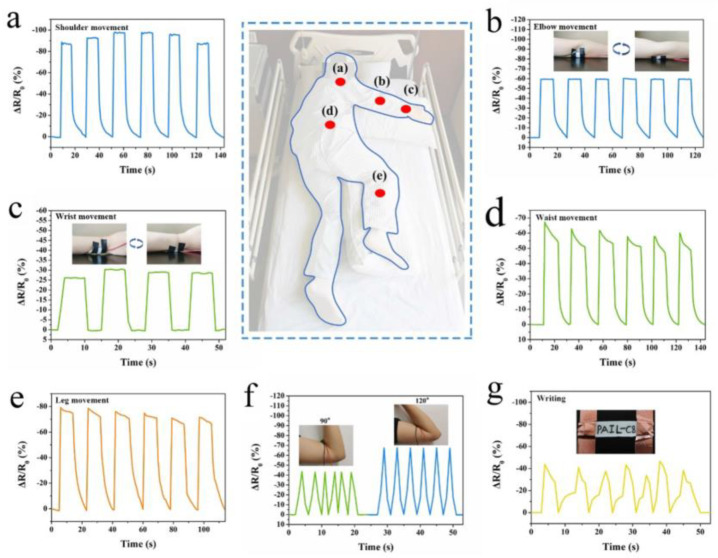
Observation of the AIL−C8 hydrogel pressure sensor for manual monitoring. Tracked movements included: (**a**) shoulder movement, (**b**) elbow movement, (**c**) wrist movement, (**d**) waist movement, (**e**) leg movement, (**f**) elbow bends at 90° and 120° angles, and (**g**) writing [39].

Dongrun Li et al. [39] utilized the antibacterial properties and electrical conductivity of imidazolidine ionic liquids to develop a polyvinyl alcohol/acrylamide ionic liquid hydrogel dressing, as depicted in Figure 14. This hydrogel demonstrated excellent pressure sensitivity, real-time responsiveness, a stable signal output, and superior mechanical properties. The integration of these features enabled the hydrogel to monitor human movement on a large scale and promptly transmit the pressure status of the patient’s wound to the nursing staff. This real-time monitoring would help to prevent secondary pressure injuries. The versatile nature of this hydrogel dressing allows for its application in chronic wound management and pressure-sensing monitoring, enhancing patient care and promoting wound healing. The toxicity of the PAIL hydrogels to L929 mouse fibroblasts was evaluated using the MTT method and the Calcein-AM/PI live/dead cell double-staining kit. During the entire incubation period, the cells proliferated rapidly, and most of the cells were spindle-shaped, without noticeable damage. The cells showed excellent survival viability (green) except for the apoptotic cells of normal metabolism (red). By observing the staining of live/dead cells with an inverted fluorescence microscope, the cell live/dead staining experiments showed that PAIL hydrogels had good biocompatibility and offered a promising material for wound dressing and pressure sensor monitoring. Huifeng Dong et al. [85] successfully developed a multifunctional hydrogel dressing to treat infected pressure ulcer wounds and monitor human health. The hydrogel dressing was created using alginate and polycation via in situ free radical polymerization and solvent displacement techniques. This hydrogel exhibited electrical responsiveness to stress, strain, and temperature. With its potential for multimodal sensing, the hydrogel demonstrated stable electrical signals for both large-scale human movements and small-amplitude movements. The multifunctional nature of these hydrogel dressings holds promising applications in promoting wound healing and monitoring human health effectively.

### 4.5. Nano-Composite Hydrogel Dressing

Polymer composite hydrogels have demonstrated extensive utilization within the realm of biomedicine, serving as materials for sustained drug release, wound dressings, and scaffolds for tissue engineering. Their prevalence can be attributed to their exceptional biocompatibility, moldability, and similarities to the extracellular matrix. Despite these advantages, their inherent mechanical limitations impede the hydrogels’ advancement. To address this issue, incorporating diverse nanoparticle variants, encompassing carbon-based, polymer-based, inorganic-based, and metal-based nanoparticles, within the hydrogel framework has emerged as a common strategy. This approach yields nanocomposite hydrogels that are characterized by their superior properties and have the ability to be tailored to specific functionalities [86,87]. In recent years, researchers have introduced nanoparticles or nanorods into hydrogel networks to obtain nanocomposite hydrogel dressings, which can not only enhance the mechanical properties, self-healing properties, and antibacterial properties of the hydrogels but can also ensure the monitoring and treatment functions of the hydrogel dressings themselves. Therefore, nanocomposite hydrogel dressings have good application prospects as biomaterials [88,89,90,91].

**Figure 15:**
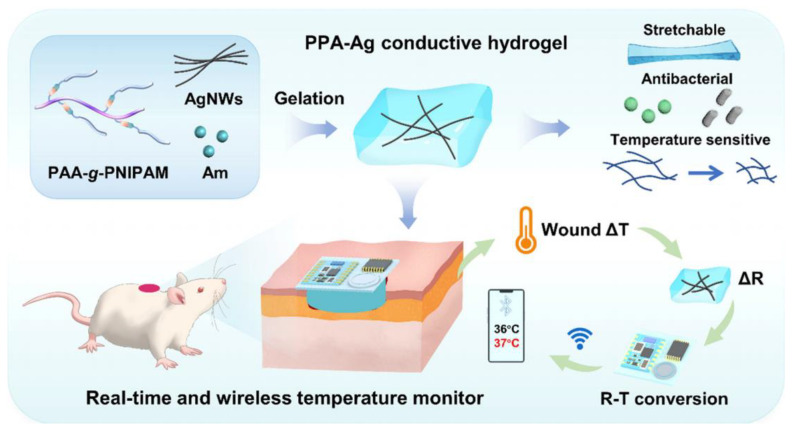
Formulation approaches for a thermo-responsive hydrogel dressing, embedded with a wireless Bluetooth module to facilitate the continuous real-time monitoring of wound temperature [92].

Ji Jiang et al. [92] designed a polymer-based wound dressing in the form of a conductive, soft, temperature-responsive, antibacterial, and biocompatible hydrogel (Figure 15), which was composed of polyacrylic acid (PAA)-grafted poly(N-isopropyl acrylamide) (PNIPAM), vinyl-based polyacrylamide (PAM) and silver nanowires (AgNWs). PAA-grafted PNIPAM acted as a conformal interface and intrinsic temperature-responsive matrix in this hydrogel dressing. PAM helped to construct semi-penetrating polymer networks (SIPNs) to improve the hydrogel’s mechanical properties. Simultaneously, incorporating silver nanowires (AgNWs) established a three-dimensional conductive hydrogel network with inherent antibacterial and sensing attributes. This engineered hydrogel framework was seamlessly integrated with a Bluetooth module, enabling the wireless transmission of temperature fluctuations to a smart device. This innovative amalgamation of a conductive hydrogel dressing with wireless transmission capabilities achieved the seamless and real-time monitoring of wound temperature. This capability would contribute to the early identification of potential infections. The demonstrated feasibility of this concept presents a compelling avenue for advancing novel approaches in enhancing wound care and facilitating advancements in various pathological diagnostics and treatments. Huiwen Pang et al. [93] developed a temperature-responsive adhesive hydrogel based on mussel-inspired dopamine chemistry and core–shell nanoparticle-regulated dynamic cross-linking. Poly (N-isopropyl acrylamide) (PNIPAM) was used as the hydrogel skeleton, endowing the hydrogel with intelligent thermal sensitivity. Core-shell nanoparticles (NPs), characterized by precise size control, were synthesized via the method of reversible addition-fragmentation chain transfer (RAFT) dispersion polymerization. These nanoparticles functioned as dynamic crosslinking cores, leading to a notable enhancement in both the adhesion and mechanical attributes of the resulting hydrogel structure.

**Figure 16:**
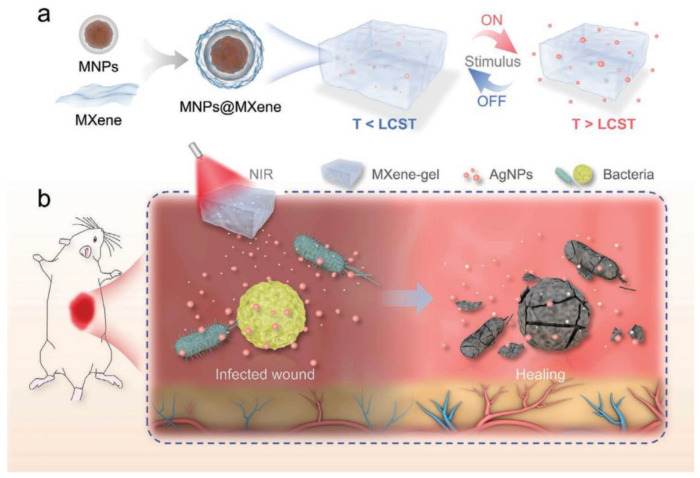
Schematic depiction of the fabrication and utilization of a responsive MXene-based hydrogel system. (**a**) The formation and drug release process of the MXene−based hydrogel system. (**b**) Deep chronic infected wound treated with NIR responsive AgNPs−loaded MXene−based hydrogel system [100].

In recent years, photothermal therapy is an area of rapid development in treating wound infection and cancers, due to its advantages of spatiotemporal control, non-invasiveness, few side effects on normal tissues, and low cost. Photoresponsive nanocomposite hydrogels have been widely used in photothermal therapy [94,95,96,97,98,99]. Xin Yang et al. [100] developed an innovative hydrogel system, based on MXene, that was engineered to possess responsive characteristics to both light and magnetism, along with the ability to control drug delivery. This design was specifically tailored to address the challenges of healing deep, chronically infected wounds (depicted in Figure 16). A novel class of intelligent drug carriers was developed by incorporating MNPs@MXene magnetic colloids into a dual-network hydrogel composed of PNIPAM and alginate. This carrier was further enriched with the inclusion of AgNPs (silver nanoparticles). The resulting MXene-based hydrogel system, which was capable of responding to specific stimuli, demonstrates the considerable potential for effectively addressing the healing of deep, chronic wounds and holds promise for use in various biomedical applications.

A summary of these hydrogel dressings with wound monitoring and treatment functions can be found in Table 1.

## 5. Commercial Hydrogel Dressings

While numerous hydrogel dressing products are currently available on the market, hydrogel dressings integrating wound monitoring and treatment functionalities have not been introduced thus far. For reference, Table 2 provides an overview of several hydrogel dressing products that are commercially available [101,102].

## 6. Conclusions and Prospects

Chronic wounds encompass various types, each exhibiting distinct characteristics that are influenced by changes in the wound microenvironment. Parameters such as temperature, pH value, blood glucose concentration, and pressure undergo fluctuations depending on the type and condition of the wound. Hydrogels, composed of hydrophilic polymers, are promising materials for constructing multifunctional platforms. These hydrogels possess several advantageous properties, including a 3D network structure, high permeability to water and oxygen, and biocompatibility. By utilizing hydrogels as substrates, it is possible to design hydrogel dressings with pH-sensitive, temperature-sensitive, glucose-sensitive, and pressure-sensitive properties. In this paper, several types of wound monitoring and therapeutic hydrogel wound dressings have been introduced in detail, including a pH-sensitive hydrogel dressing, thermo-sensitive hydrogel dressing, blood-glucose sensitive hydrogel dressing, pressure-sensitive hydrogel dressing, and nano-composite hydrogel dressing. These hydrogel dressings with wound-monitoring functions can also facilitate treatment, based on the monitoring results.

Nonetheless, the development of wound monitoring and therapeutic hydrogel dressings presents several significant challenges. Firstly, enhancing the precision of monitoring hydrogel dressings for parameters such as pH, temperature, and blood glucose is imperative. Achieving greater accuracy in these aspects is crucial. Furthermore, the real-time acquisition of dynamic data from monitored wounds poses a time-sensitive challenge. An instance of this is the use of hydrogel dressings employing pH-sensitive dyes to gauge wound microenvironment changes; measurements such as alterations in color could be susceptible to interference from wound exudate, thereby affecting monitoring precision. Secondly, the biocompatibility of hydrogel dressings must be meticulously addressed. Notably, the nanoscale constituents utilized in nanocomposite hydrogel dressings, such as nanoparticles, necessitate thorough scrutiny. Although these nanomaterials impart remarkable antibacterial attributes and facilitate controlled drug release, concerns arise regarding the potential impact of releasing nanoparticles into the human circulatory system via wound sites. The accumulation of nanoparticles in the body might trigger adverse reactions such as blood clot formation. Lastly, the commercialization of hydrogel dressings that are embedded with wound monitoring and treatment capabilities represents another formidable hurdle. The utilization of newly designed materials in the majority of contemporary hydrogel dressings necessitates sustained testing prior to market launch. Furthermore, many wound-monitoring hydrogel dressings are still in the experimental phase. The majority of these products are disposable, and their journey toward commercialization is lengthy and intricate. Addressing these multifaceted challenges requires interdisciplinary collaboration, rigorous testing, and continual innovation to ultimately realize the successful integration of wound monitoring and therapeutic functionalities into hydrogel dressings on a commercial scale.

The future prospects regarding hydrogel dressings with wound monitoring and treatment functions can be summarized as follows:

1. The development of multifunctional hydrogel dressings is promising. For example, hydrogel dressings can monitor the wound microenvironment and also have excellent antibacterial, anti-inflammatory, antibleeding, mechanical properties, injectable, and self-healing properties;
2. The need for the preparation of hydrogel dressings that can meet all the requirements in the whole process of wound healing is urgent. Since the wound repair process is complex and involves dynamic changes in various parameters, providing functionality on demand is a direction for further research;
3. The integration of wound microenvironment monitoring and telemedicine is an important direction. It is of great interest to develop a hydrogel wound dressing that can simultaneously monitor the state of the wound’s microenvironment and inform the physician. A hydrogel dressing with wound monitoring and treatment functions can guide doctors to remotely control the treatment of wounds, which may be the future trend.

## Figures and Tables

**Table 1:** Hydrogel dressings with wound monitoring and treatment functions for different chronic wound types and their advantages and limitations.

Types of Chronic Wound	Types of Monitoring	Monitoring and Treatment Components	Advantages	Limitations	Ref.
Infected wound	pH	Brilliant Yellow, cabbage juice, and gentamicin-loaded alginate fibers	Offer comprehensive monitoring and treatment based on accurate and timely wound information	The exudate itself may be affected by color	[36]
	pH	Litmus, convolutional neural network	Prevent or reduce the risk of bacterial infection	The wound cannot be treated accurately, and the solvent replacement method used in the preparation of hydrogels is time-consuming	[73]
	pH	Bromothymol blue, near-infrared absorption conjugated polymers, chitosan	In situ visual diagnosis of bacterial infection and photothermal therapy	Easy leaching of dyes	[38]
	pH	Curcuma longa extract	Visual detection, antibacterial	Inaccurate monitoring ability, easy leaching of dye, and poor wound healing effect	[74]
	Temperature	Temperature sensor and four UV-LEDs, gentamicin	Flexibility, compatibility, high monitoring sensitivity, and durability	High cost, short service life of response elements, and can only be used as disposable dressings	[81]
	pH, temperature	Potentiometric pH sensors, NIPAM	Dual response of pH and temperature, precise monitoring, and treatment	High-cost and complex preparation	[82]
Burn wound	Temperature	Silver nanoparticles	Excellent antibacterial activity	Cannot monitor the wound microenvironment	[78]
	Temperature	NIPAM, diclofenac sodium	Controlled and targeted drug delivery	Inaccurate perception of temperature changes	[80]
Diabetic wound	pH, glucose	Schiff base, phenyl borate base, insulin, fibroblasts	Effective control of blood glucose levels treats diabetic wounds	The rate of insulin release is not well controlled	[84]
	pH, glucose	Phenyl borate ester bond, metformin, copper-loaded dopamine nanoparticles	pH and glucose responses to controlled treatment, self-healing, good injectability, and adhesive properties	The rate of metformin release is not well controlled	[40]
	pH, glucose	Phenol red, glucose oxidase (GOx), horseradish peroxidase	Effectively monitor pH and glucose changes	Horseradish peroxidase is needed	[37]
	Temperature, glucose	NIPAM, methyl acrylamide phenyl boric acid	Continuous real-time monitoring and differentiation of temperature, glucose concentration, and wound strain	Inaccurate perception of temperature changes	[69]
Pressure ulcers	Pressure	Imidazolidine ionic liquids, polyvinyl alcohol, acrylamide	Excellent pressure sensitivity, real-time responsiveness, stable signal output, and superior mechanical properties	Complex preparation process	[39]
	Pressure	2-(methacryloyloxy)-N,N,N-trimethyl ethylamine chloride, dopamine hydrochloride	Effectively monitor human health	The solvent displacement method is time-consuming	[85]

**Table 2:** Some commercially available products incorporating hydrogel-based dressings.

Hydrogel	Company	City and Country	Main Constituent
Algisite M	Smith & Nephew	London, United Kingdom	Alginate
Amniomatrix^®^4	Derma Sciences Inc.	Pennsylvania, United States	Amniotic membrane and fluid constituents
Comfeel^®^ Plus Contour Dressing	Coloplast Corp.	Guangdong, China	Carboxymethylcellulose
CovaWound^™^ Hydrocolloid dressing	Covalon Technologies, Ltd.	Georgia, United States	Hydrocolloids
Cutimed^®^ Gel	Bsn Medical Gmbh	Hamburg, Germany	Carbomer 940
DermaFilm^®^	DermaRite Industries, LLC	New Jersey, United States	Hydrocolloids
Helix3-cm^®^	Amerx Health Care Corp.	Florida, United States	Collagen
Inadine^™^	Systagenix	Nevada, United States	Polyethylene Glycol
Kaltostat^®^	Convatec	Shanghai, China	Alginate
Kendall^™^ Hydrogel Dressing	Cardinal Health	Ohio, United States	Glycerin formulation
Sofargel	Sofar	Guangdong, China	Carbopol 974P
Tegaderm^™^ Hydrocolloid Dressing	3 M Health Care	Leicestershire, United Kingdom	Hydrocolloids

## Data Availability

No new data were created.
